# Imaginaries of Dying: The Visual Rhetoric of Stock Images Tagged with “Palliative Care”

**DOI:** 10.1177/00302228231184296

**Published:** 2023-06-27

**Authors:** Gaudenz Urs Metzger, Tina Braun

**Affiliations:** 1 Department Design Trends & Identity, 31029 Zurich University of the Arts, Zurich, Switzerland; 2 Institute of Design Research IDR, 30337 University of the Arts Bern, Bern, Switzerland

**Keywords:** stock photography, palliative care, hospice philosophy, dying and death, visual rhetoric

## Abstract

In digital culture and its global economy, images circulate transnationally and shape cultural ideas about social and existential issues. While there is growing interest in death online, few studies have investigated the role of visual material in different forms of communication in this field. In this article, we examine the depiction of dying and death in stock photographs tagged with “palliative care” drawing on an image corpus of 618 photographs. Stock photographs are images produced for commercial purposes that are stored in databases by agencies on the Internet. To analyze how these representations depict fictional palliative care settings, we used visual grounded theory. The findings show that typical caregivers are portrayed as emphatic individuals, while patients appear as composed human beings facing death without fear. We argue that the images represent aspects of the modern hospice philosophy and the cultural narrative of healthy aging.

## Introduction

Since the 1990s, individual and collective negotiations of illness, dying, and death have become increasingly visible in the public sphere through various media and in both verbal and visual communication. Thomas Macho and Kristin Marek (2007) have pointed to the “new visibility of death” and shown how media and the arts counteract the modern sequestration of death and make this (taboo) topic the subject of lively societal debate. Other authors have emphasized that digitalization is changing the way Western societies deal with existential givens, such as illness, loss and death (Andersson, 2019; Arnold et al., 2018; Caduff, 2022; Lagerkvist & Andersson, 2017; Walter et al., 2012; Walter, 2020). In recent years, the Internet and social media have become important components of a new culture of death in which images as vehicles of meaning, emotions, values, and knowledge are becoming increasingly important alongside verbal communication. Up to now, however, death studies have paid little attention to the “pictorial turn” (Mitchell, 1992, 1994), which affects both the practices of mourners and dying people (Gibbs et al., 2015; Metzger, 2023a) as well as communications produced by health care providers. Today, hospitals and outpatient services advertise their palliative treatments and therapies through different online and offline media channels using a variety of visual materials. In this paper, we aim to contribute to this emerging field of study by analyzing commercial stock images tagged with “palliative care” found in the online catalogues of four large image agencies. These images, which circulate globally on the Internet, have not been explored to date and are utilized, for example, on websites to advertise hospice and palliative care. In this article, we will demonstrate that the examined stock photographs articulate values and norms of a humane, patient-centered end-of-life care that addresses the physical, emotional, spiritual, and social needs of patients according to the ideals of the modern hospice movement (Clark, 1998, 2018; Graven & Timm, 2021; Hart et al., 1998; Metzger, 2023c; Randall & Downie, 2006). Cicely Saunders—the founding figure of this movement—herself used the medium of photography to document positive emotional states among dying patients and to promote her concept of terminal care and its associated ideals, practices, and values (Saunders, 1965, 1972). Criticizing the “medicalization”^1^ of death, Saunders offered a new care paradigm of how “this event might be viewed, managed and celebrated within a new psychological landscape” (Nash, 2013, p. 129). As we will show, the analyzed images can be located within this Western tradition of dealing with dying and death and its particular kinds of discourses, such as, for example, that of spirituality (Walter, 1994, 2002). They portray a vision of a “good death” in which death appears as a gentle farewell and the possibility of religious transcendence, while individual nuances and experiences of dying are blanked out, especially those that are negative, such as grief or despair. This can be problematic as it might create false expectations among patients and relatives about the last phase of life. Furthermore, we argue that these images do not represent dying, but the desire for healthy aging.

Our analysis is based on a large corpus of commercial stock photographs tagged with the keywords “palliative care” that can be found in the online catalogs of the agencies *Gettyimages*, *shutterstock*, *iStock*, and *Adobe Stock*. Editorial stock images, that are used, for example, in newspapers to document events related to dying and death, were not included in the corpus. Our focus is on photographic representations that depict fictional palliative care settings, as we will further elucidate in the methods section. We regard these photographs as digital artifacts that offer a vehicle for understanding the social world. According to Manfred Lueger and Ulrike Froschauer (2008), artifacts include all objects that are anchored in the material world and “have been created, handled, modified, or transformed by human intervention” (p. 11, author’s translation). Digital photographs, as two-dimensional artifacts, are excellent tools for gaining insights into a society’s cultural beliefs about dying and death. As photographs and other forms of pictorial representation do not merely depict objects but exemplify perspectives that have been formed culturally (Wiesing, 2007), they mirror the ideas, values, and narratives of both individuals and groups. In today’s digital culture, where photographs are constantly being taken, shared and stored on the Internet, large amounts of data have become accessible to researchers, opening up new sources for understanding practices related to dying and death. In this article, we take this opportunity to examine how palliative care and its ideas and values are represented in commercial stock images.

## Stock Photography: History and Production

Stock photos are images that are produced and stored by agencies in databases for future commercial use; they are classified according to classical topics such as “nature,” “leisure,” or “sports,” as well as current topics such as “portraits of diversity” or “stand up for pride.” Photo agencies have been in existence since the beginning of the 20^th^ century (Blaschke, 2016). In recent decades, however, digitization has brought about a significant change in the market. A myriad of images can now be found and purchased easily in catalogs that are accessible via the Internet. Bill Gates and Mark Getty played a fundamental role in this development by investing in the digitalization of archives. Gates founded the *Corbis* agency in 1989, while Getty founded *Getty Images* in 1995. *Corbis* owns the valuable Bettmann Archive (among others), which is a unique historical collection of approximately 11 million images. From the moment they were founded, both *Corbis* and *Getty Images* have bought up numerous archives and agencies and have been producing digital copies of historical photographs. Gates and Getty became the leaders on the global image market. Their declared goal was to produce images strictly in line with demand and to sell these images worldwide (Ullrich, 2008). In addition to these two large-scale providers, so-called “microstock sites” are also increasingly popular on the Internet today. These sites are primarily supplied by amateurs and are notable for their relatively low price levels.

Overall, digitization profoundly changed the conditions for the production of photographs and the possibilities for their reproduction, as well as their different modes of reception and archiving practices. The technical reproducibility of artwork and images was made possible in the early 19th century by the invention of the photographic process that had a decisive impact on the perceptual reorganization of modern culture (Benjamin, 1935/1969). From the 1980s onward, digital methods expanded these possibilities and allowed individuals to reproduce photographs with a few clicks of a mouse and to store them in electronic archives. Today, photographs can be mixed with text, graphics, audio, film, etc., and have metadata inscribed directly into them. The semiotics of photography in the digital age resembles that of other forms of visual representation (Lunenfeld, 2000; Mitchell, 1994), yet the traditional function of the photo archive has remained the same: it transforms what is specific and idiosyncratic about a picture into something typical and emblematic (Sekula, 1986). Unlike in the past though, where the task of producing such catalog data used to be assigned to professionals, today it can be undertaken by amateurs and even by intelligent machines working with pattern-recognizing algorithms. In this way, images circulating on the Internet, such as stock photographs, are made accessible and retrievable, but even more importantly, the metadata inscription culturally codes phenomena such as palliative care and defines what is a typical instantiation of it.

Borrowing a concept from the French Philosopher Jacques Rancière, we argue that stock photos as hybrid text-image-assemblages establish a “distribution of the sensible” that influences what is visible and invisible as well as sayable and unsayable in a society about a topic, such as dying and death. According to Rancière (2004), the “distribution of the sensible” are systems that steer our perception and disclose “the existence of something in common” (p. 13), while at the same time excluding alternative views and perspectives.^2^ The stock photos examined in this article, as we will see, are designed based on common Western assumptions of a “good death”. They reduce the sensitive topic to a handful of motives and thus influence how it is imagined. A crucial factor in this narrowing down of motives, to which we will now turn, can be seen in the economy of production of this specific image type.

The way in which commercial stock photos are produced provides an important key to understanding their function and aesthetics. In contrast to editorial images that document the details of a situation or event, commercial stock photos are intended to be as non-specific as possible. This relates to the economic premise of their reusability, as Wolfgang Ullrich has noted:

*Apart from their open context, it is important that a stock photo should show nothing that can be dated precisely or might have only a short half-life. Seasonal fashion accessories or distinctive hairstyles are therefore not desirable; ideally, nor should the design of a cell phone be precisely recognizable. *(Ullrich, 2008*, *p. 53, author’s translation)

In addition, according to Ullrich, stock photos should not violate any taboos of the cultures that are their largest markets (USA, Europe, East Asia, etc.), such as nudity or certain political gestures or topics. Stylistic elements such as blurring or a narrow perspective are often utilized to realize the economic premise of reusability. As a result, stock images often appear generic and lack idiosyncrasies. What might be an aesthetic shortcoming on the one hand is in fact an economic advantage on the other. The images that are especially marketable across the world are those that express as few cultural idiosyncrasies as possible. For this reason, Ullrich calls stock images “pictures to forget” that allow for little or no personal references:

*Since they [stock images, author’s note] lack any anecdotal impact, and since even the models in them are selected so that their faces look as average as possible and possess few distinctive characteristics, it is hardly possible for the viewer to associate stock photos with their own experiences and thereby to remember them: these images do not allow one to feel any personal relationship towards them. *(Ullrich, 2008*, *p. 57, author’s translation)

This market orientation prevents visual idiosyncrasies and makes commercial stock images particularly well suited for purposes of idealization and for expressing stereotypes. These images have to appear relatively realistic in order to achieve their communicative goal, but ultimately they do not attempt to fulfil documentary photography’s promise of authenticity (see e.g., Barthes, 1981; Talbot, 1844–1846/2011; Walton, 1984). Paul Frosh has argued that commercial stock images primarily operate in the register of the symbolic and the rhetorical:

*[…] stock images are not usually governed by a rigorous realism and empiricist code that presume and emphasize their “truth value”, their fidelity to the depicted reality […]. In other words, the modus operandi of stock images is overtly symbolic rather than documentary: their master discourse is rhetoric, not science. *(Frosh, 2003, p. 99)

Embedding stock images in communication contexts can intensify their symbolic meaning and their rhetorical function, with the latter being intended to influence the viewer’s attitudes and convictions in a manner that is both immediate and affective (Sachs-Hombach & Masuch, 2007). Such contexts, for example, could include the website of a hospital that is advertising its palliative care services. The images be aimed at convincing potential patients and their relatives that one can die there in good hands and that dying people are not “banished behind the scenes” (Elias, 1982/2001, p. 11), as was often the case in modernity’s death-defying culture. Although the modern hospice philosophy reacted against modernity’s sequestration of death (Thoresen, 2003) and tried to tackle its problems, this fear still seems present in people’s minds. A recent study by the Berlin Institute and the Körber Foundation found that one core unease of the respondents was that nursing staff have too little time, and that one ultimately dies alone (Carrasco Heiermann et al., 2020). As we will show, it is precisely such fundamental fears that stock images endeavor to address and ameliorate.

## Image Corpus and Analysis Method

Our analysis is based on an image corpus of 618 stock photos. In order to obtain a comprehensive overview of the stock images available, we searched through four principal stock agencies, namely *Gettyimages*, *shutterstock*, *iStock* and *Adobe Stock,* using the keywords “palliative care”. Similar search terms such as “end-of-life care,” “end of life,” and “dying” were excluded so that we were able to focus as closely as possible on the visual representation of palliative care. When collecting the material, our focus was on commercial stock images that stage palliative care situations or settings, although the boundaries between staged photography and documentary photography are not clear-cut.^3^ As mentioned above, we did not include editorial images of deathbed settings, nor illustrations or pictograms in our collection of materials. Our research interest was in how photography is employed as a means to create fictional palliative care realities that convey cultural ideas and norms. In principle, we tried to achieve a degree of completeness and representativeness by searching through four main stock agencies, although we did not include mircrostock sites.

### Visual Grounded Theory

Our analysis of the visual material was undertaken in line with the methodological principles of visual grounded theory (Grittmann, 2018). Elke Grittmann (2018) highlights that grounded theory—developed by Glaser and Strauss (1967)—offers distinct principles and procedures for the collection and interpretation of different types of data that enable researchers to better understand complex phenomena. The open-coding method can be used to examine social worlds and the fabric of practices, narratives, values, artifacts, media etc., that constitute them. According to Grittmann, 2018, (pp. 195–197) the aim of visual grounded theory is to reconstruct recurring image motifs that are similar to each another, and that provide information about cultural constructions, for example gender, identity or religious ideas. Such image motifs can be discovered utilizing the coding technique of grounded theory, which consists of the constant comparison and grouping of data. The ultimate goal of developing these types is to uncover connections between the sociocultural context and the representation of the phenomenon—that is, to find the cultural patterns and narratives out of which it originated.

**Figure 1.**
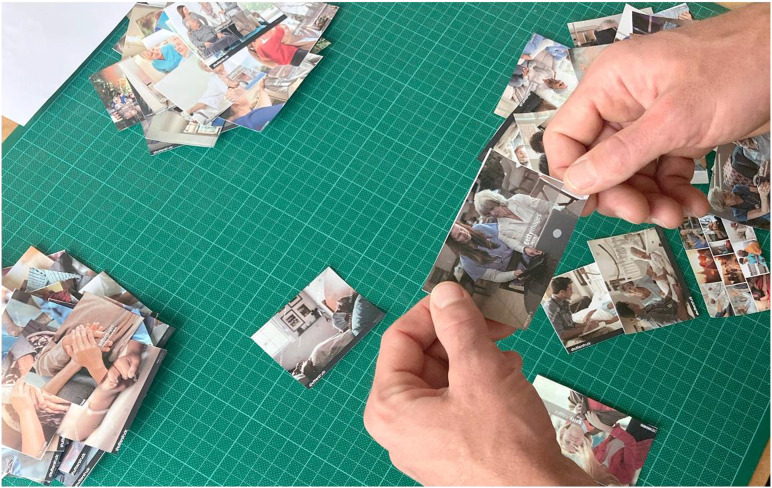
Laying out and arranging the photographs.

In an initial step, we sifted through the images and organized them into different groups by looking for thematic similarities among them. In line with grounded theory (Corbin & Strauss, 2008) we began our analysis with open coding that identified actors, interactions, and phenomena in the stock images. To do this, we printed the photographs as thumbnails and laid them out on tables (Figure 1). In the iterative research process, we combined the tasks of selection, analysis, and interpretation; we subsequently employed axial and selective coding to group images with similar themes and finally to arrive at concepts and categories. This enabled us to identify typical representations of palliative care in commercial stock images that we have labeled as follows: (1) presence; (2) composure; (3) empathy; (4) transcendence. However, these categories also overlap and their boundaries are not always clear-cut. In the following, we present these four representations of palliative care using exemplary image tableaus and image descriptions that illuminate the rhetorical and stylistic devices utilized in these photographs. These stylistic devices include, for example, color moods, perspective, cropping and sharpness/blurring.

## Imaginaries of Dying

### Presence

**Figure 2.**
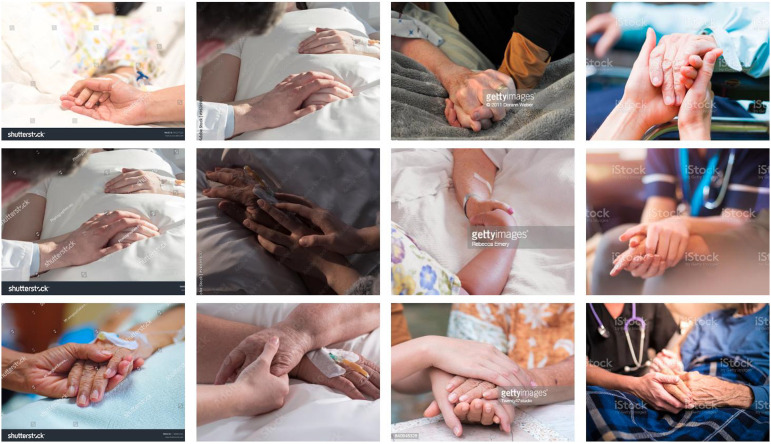
Presence.

The subject that is by far the most common in our image collection is “holding hands.” These photographs have been tightly cropped, with the focus on interlocked hands and fingers; occasionally, part of an arm is also visible. The patients are recognizable by details such as cannulas and plasters on their arm, while the nursing staff are characterized by items such as their clothing (surgical gowns) and medical instruments (stethoscopes). Often, the shallow depth of field means that these details are only hinted at, and are reduced to a minimum in accordance with the above-mentioned economy of production. No faces are shown. The context of “illness/dying” is communicated visually through bed sheets and blankets and by the parts of bed frames, arm grips, or wheelchairs that are only just visible. Some photographs also allow us to draw conclusions about the type of people on site—such as when someone is sitting on a chair next to a patient and holding their hand. These images depict the motif of holding hands in a very similar way, and they all suggest that we do not die alone and that someone is there for us in what is perhaps the most difficult moment of our life. This desire is given rhetorical support by the tight cropping of the image detail, directing the viewer’s gaze to tightly entwined fingers symbolizing human attachment, warmth, and the presence of a fellow human being at the end of life. Another rhetorical device found in these photographs is the use of fabrics. The hands are depicted as resting on soft materials such as terry toweling and bed coverings, emphasizing feelings of comfort and being cared for. This interplay between perspective, materiality, and the motif of holding hands accentuates close, physical bonds in order to construct an imagination that provides a human face to the end of life and even to death itself. We have assigned this image type the category “presence” (Figure 2).

### Composure

**Figure 3.**
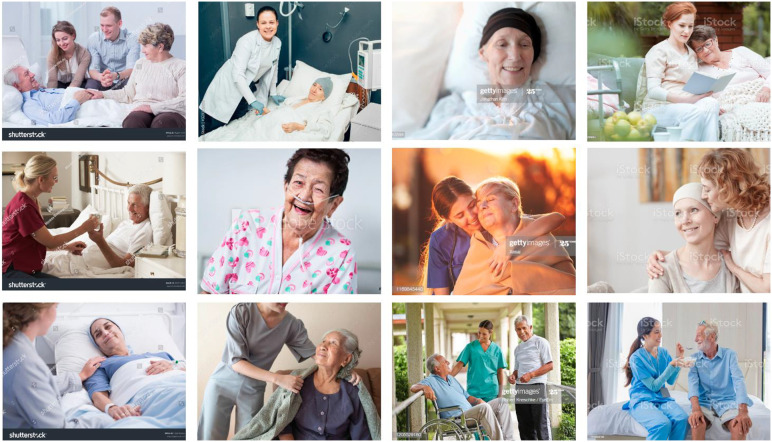
Composure.

The second theme we identified is “composure.” Numerous photographs depict portraits of contented, relaxed, smiling patients who are being cared for lovingly by relatives or nursing staff. The actions depicted here include, for example, a nurse bringing a patient a cup of tea, or placing a woolen blanket around them. During these activities, the nurses and the patients exchange friendly glances; the eyes of the latter seem to express gratitude. All those depicted have bright smiles on their faces. Topics such as grief, fear, or despair—which are all part of the dying process—are omitted here. These images express our desire for humane care when dying and a painless, contented farewell to life. This idea is reinforced by the use of light as a stylistic feature. Warm hues—such as a background sunset—emphasize the intended visual impact that aims to convince the viewer of the loving, caring atmosphere to be found in a palliative care setting. In addition, these staged scenes are brightly lit, which serves to mitigate any sense of fear that the topics of dying and death might convey, given that they often have negative social connotations. By contrast, dark and cold moods invoked by blue tones—which are often utilized in depictions of surgical and medical practices—are absent from these images. Furthermore, as in the photographs discussed above, the message intended by the image is underscored by the textiles depicted. The patients are enveloped in bright white blankets and are wearing dignified shirts, T-shirts or blouses. The context of medical care is largely obscured from view. The care scenes are often staged in settings close to nature, such as a garden or an immaculate home. This prevents the viewer from being reminded that death might be associated with machines or the cool rationality of modern medicine. The images instead suggest that dying is a peaceful, painless process in an aesthetic environment—one in which we can face death with composure (Figure 3).

### Empathy

**Figure 4.**
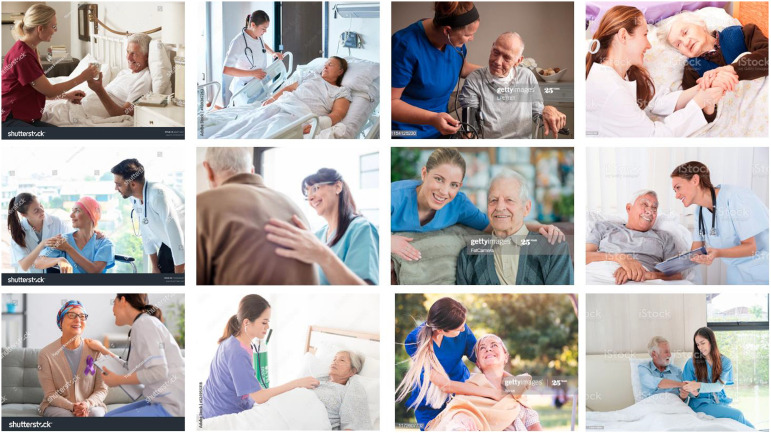
Empathy.

The third category, which we call “empathy,” concerns the construction of typical nursing staff in palliative care by means of recurring image motifs portraying interactions, actions and gestures. The focus of these photographs is entirely on the patients as individuals. They are being cared for by predominantly young, slim, good-looking women. By contrast, the patients depicted are elderly, predominantly white people. This contrast constructed between old and young infers that the dying are primarily people of advanced age. Furthermore, they are not depicted as being in a poor physical condition, marked by severe illness; instead, they appear fit and healthy, perpetuating the socially entrenched idea of successful and healthy aging. This image category depicts both medical activities (taking a pulse, admission consultations, taking a case history, etc.,) and empathetic, bodily gestures and actions. One important rhetorical device that is employed in these stock photographs is the selection of an image detail that directs the viewer’s attention to the interaction between nursing staff and patients. In contrast to the close-up images of hands discussed above, the space where the action takes place in these scenes is openly revealed. This enables viewers to orient themselves in the space shown, and to contextualize the motifs that are depicted. The medical setting in which the interactions between the image subjects are taking place is immediately recognizable, such as a patient’s room or a consultation room. What is striking about these depictions, however, is that the nursing staff have often placed an arm around the patient’s shoulders while the actions are being undertaken. This caring gesture signifies that the care work being performed is considered less a professional medical activity then an ethical task or vocation being conducted with human sensitivity and compassion. These stock photographs construct the idea of typical palliative care staff as deeply empathic people who respond to a patient’s situation and needs with patience, understanding, and sensitivity (Figure 4).

### Transcendence

**Figure 5.**
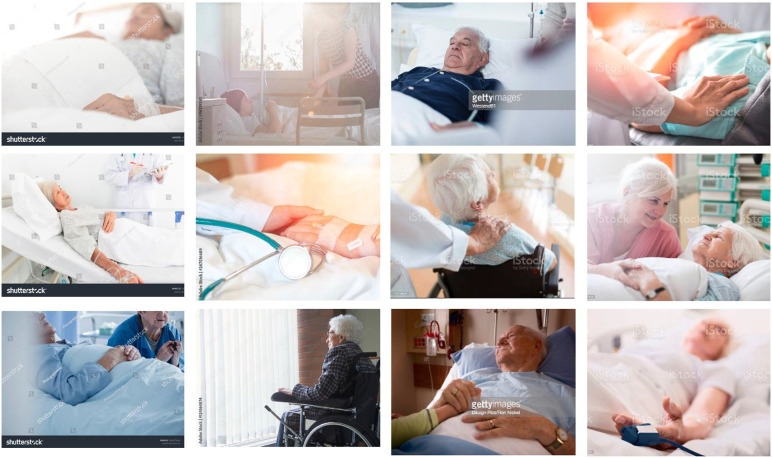
Transcendence.

The fourth category that emerged from our analysis constitutes the “transcendence” image type. These photographs show people lying in bed with their eyes closed, or sitting in a wheelchair while looking out of the window, their back to the viewer. The patients are supported in their difficult situation by nursing staff or relatives who are often depicted holding their hands in a caring manner. A core rhetorical, stylistic device used in these images is bright light: it veils the people depicted in a peaceful, contemplative atmosphere. Rays of light enter the images obliquely from above, allowing a gentle expression to appear on the faces of the dying, who already seem to be in a state of transition to another plane. Other design elements that underscore this idea include the slight blurriness as well as the bands of white that are superimposed on some of the photographs. When combined, these stylistic devices, as well as the imagery chosen, construct death as a transcendent experience and possible transition into a life in another world. They suggest that death might not be an irrevocable extinction of existence, but instead the crossing of a boundary and life’s completion. In this way, these stock photographs create a religious or spiritual image of dying and death, with metaphors and symbols of light suggesting something auratic, mystical, transcendent, and divine (Figure 5).

## Discussion and Conclusion

In the stock photographs we have analyzed, we see patients, their relatives and nursing staff, all of them acting at a far remove from any painful, sad, or stressful events that might characterize everyday life in palliative care. The categories and topics presented here offer a glimpse into desires and wishes that may be associated with the end of life, such as dying within one’s family circle or a painless death. If we compare these narratives to those reported by the dying themselves (see, e.g., Caduff, 2022; Kellehear, 1990, 2015; Lawton, 2000; Menzfeld, 2018; Metzger, 2023b), we can observe how many of the core topics highlighted by these authors, such as uncertainty, the fear of dying and of death, loneliness, and the ailing and decaying body, have simply been omitted from the imagery. Thereby, the studied stock photographs perpetuate Western narratives of a “good death” and healthy aging, “which is the prevailing ideology of health care professionals” (Kellehear, 2023, p. 145), along with gender stereotypes in care work. They depict palliative care situations suitable for advertising purposes, in which dying—contrary to the modern hospice movements’ idea of accepting death as a natural process and fact (Hart et al., 1998)––is repressed. Health care institutions are presented in these images as well-functioning, spotlessly clean places of care, populated by young, primarily female caregivers who look after their mostly fit patients with a sense of sacrifice and devotion. The rhetorical, stylistic devices used in the images help the viewer block out feelings of anxiety and insecurity which might arise in the confrontation with illness and death. This arises primarily through the generic visualization of the settings and the protagonists, who are depicted as devoid of any individual identity. Furthermore, these rhetorical devices support the reusability of the images by utilizing selective cropping to focus on details that offer as little context as possible. The actions depicted are similarly non-specific, and also mean that these images become transferable to other healthcare providers that are not specialized in palliative care, but are also potential purchasers of these photographs.

As the findings show, the notion of a “good death” that is promoted by these images is closely linked to the values, beliefs, and practices of the modern hospice philosophy as initially laid down by Saunders’ work with terminally ill people at St Christopher’s Hospice in London in the 1960s and 1970s. Saunders’ “potent blend of secular help and spiritual comfort” (Nash, 2013, p. 130) aimed to help patients to go through this difficult situation and eventually experience fulfillment and catharsis at the end of life. As mentioned at the outset, to promote this ideal, Saunders herself published numerous photographs and sketches. Our analysis revealed that commercial stock photographs of palliative care convey this same ideal by depicting patients as individuals who seemed to have worked through grief and anxiety and face impending death with composure and contentedness.

The categories of “presence” and “empathy” allow us to observe a further connection between the examined stock images and the ethical principles of the modern hospice philosophy. In order to assist people in the existential situation of dying and to protect them from loss of meaning and despair, Saunders developed the concept “Watch with me.” The concept combines empathy, emotional sensitivity and physical presence with analytical observation, and can be regarded as a central pillar of modern hospice care (Saunders, 2003, foreword by Clark). For Saunders, care is primarily grounded in the idea that humans are inherently responsive and relational beings, a premise her work shares with moral theories known as *ethics of care* (Metzger, 2023c). As we have shown, sensitivity and compassion towards patients are core visual messages in the examined stock images. They do not primarily depict palliative care as a medical activity, but rather position it as a genuinely human task and vocation that depends on the possibility and willingness of nursing staff to establish an emotional bond with the dying.

In addition, our analysis demonstrates that the analyzed stock photos construct a religious/spiritual conception of dying and death that operates along the distinction of immanence and transcendence. Since the rise of humanistic values during the Renaissance––and especially since the secularization and modernization of Western societies––the practices dealing with dying and death have become highly individualized, while collective Christian rituals have declined (Elias, 2001, 1982/2001; Giddens, 1991; Mellor & Shilling, 1993). In palliative care, this development is reflected in a preference for subject-oriented concepts of spirituality above those of traditional religion (Walter, 2002). The studied images, however, allow for different readings of transcendence: transcendence as self-transcendence in the sense of spiritual growth and healing at the end of life or transcendence as a path to a post-physical existence in the hereafter. Either way, the idea of transcendence is reinforced by the metaphors and symbolism of light. In order for these stock photographs to be globally distributable and deployable as advertising materials in palliative care contexts across different cultures, they may not show any specific religious symbols such as a crucifix. Instead, what is mystical, transcendent, and divine is implied visually by the element of light, which is a medium and symbol common to many religions (Mohr, 1999). The light metaphors, along with the motif of the window, which in Romantic aesthetics signifies the metaphorical threshold between what is inner and outer, tangible and intangible, or finite and infinite (Rickenbacher, 2015), create a conventionalized notion of dying and death as a religious or spiritual moment of transformation, based on a polar division of the world into the opposing realms of the profane and the sacred.

To conclude, we want to emphasize that stock photography of palliative care paints a highly idealized and euphemistic picture of dying and death. The stereotypical narrowing down of the imagery to a few motifs such as “holding hands” or “composure” means that the individual nuances and experiences of dying are erased, especially those that are negative, such as grief or despair. As a result, these images infer that the final phase of life and dying is something peaceful and entirely unproblematic. This can possibly raise false expectations and hopes among patients and relatives and subsequently may also trigger conflicts with institutions and members of staff if the dying process does not proceed according to this ideal. More empirical research is needed on how these images are perceived by those affected and what ideas about dying and a stay in a hospital or hospice they evoke in them. In any case, we know from our conversations with palliative care nurses that they are very critical of the representation of their work in these types of images and that they would appreciate more versatile and realistic visual representations of palliative care. For them, the images represent an unattainable ideal of care that clashes with everyday life and work in the clinic. It can thus be assumed that the communicative goal of these photos, that is, to soothe fears and worries related to the end of life, can also have negative impacts in settings of dying. We argue that behind the examined stock photographs lurks an attempt to “tame death” that glosses over this difficult topic and tries to make it socially acceptable and amenable to portrayal in the mass media and advertising.^4^ Thus, the “distribution of the sensible” that stock images of palliative care establish is something that we believe should be regarded with a highly critical eye. These images do not provoke or frighten, as documentary representations of dying and death might do. Their potential for harm lies elsewhere—namely in their evocation of illusory ideas about the final phase of life and about the possibilities of palliative care treatment.

## Notes

1. Medicalization is defined as “the process by which previously nonmedical problems become defined and treated as medical problems, usually as diseases or disorders” (Conrad, 2013, p. 196).

2. For Rancière, aesthetics and politics cannot be separated. He introduces the notion of the distribution of the sensible to show how art and aesthetics regulate and determine identities, bodies, participation and exclusion, and positions of speech.

3. Abigail Solmon-Godeau (1991) argues that documentary photography is a historical category, not an ontological one. Nevertheless, it is useful for differentiating between different uses for photography.

4. In his romanticized account of the Western history of death, the French historian Philipp Ariès (1977/2008) differentiates between a pre-modern “tamed death” and modern death that has become “wild”. Ariès’ claim is that death has become “wild” in modern times because it has been excluded from society and has become an object of academic medicine. However, he fails to explain why the social embedding of death in a community or religious rituals necessarily facilitates dying, while modern forms of coping with this process would generally endanger the possibility of a “good death.”
